# Risk Factors for Progression of CKD with and without Diabetes

**DOI:** 10.1155/2022/9613062

**Published:** 2022-08-22

**Authors:** Xiaohong Zhang, Yuan Fang, Zhenhuan Zou, Pianpian Hong, Yongjie Zhuo, Yanfang Xu, Jianxin Wan

**Affiliations:** ^1^Department of Nephrology, Blood Purification Research Center, The First Affiliated Hospital, Fujian Medical University, Fuzhou 350005, China; ^2^Fujian Clinical Research Center for Metabolic Chronic Kidney Disease, The First Affiliated Hospital, Fujian Medical University, Fuzhou 350005, China

## Abstract

**Objective:**

We aim to identify independent risk factors to predict CKD progression to end stage renal disease (ESRD) in patients with or without diabetes.

**Methods:**

In this retrospective study, we enrolled CKD stage 3-4 patients between January 2013 and December 2018 and followed them until December 2020 or the initiation of dialysis. We used Kaplan-Meier to plot the survival curve. Univariate and multivariable Cox proportional hazards model was used to explore risk factors affecting the progression of CKD. The final model was used to construct nomogram for predicting CKD progression. Calibration plots and concordance index (C-index) were used to evaluate the accuracy and discrimination of the risk model.

**Results:**

We enrolled 309 CKD patients, including 80 cases in G3a, 98 cases in G3b, and 131 cases in G4. Among them, 141 patients had diabetes and 168 did not. The mean age of patients at enrolled was 57.86 ± 15.10 years, and 67% were male. The median follow-up time was 25.6 months. There were 81 patients (26.2%) that started dialysis in the total CKD cohort, 52 cases (36.9%) in the CKD with diabetes group, and 29 cases (17.3%) in the CKD without diabetes group. Hypoalbuminemia (*HR* =2.655, *P* < 0.001), proteinuria (*HR* =2.592, *P* = 0.042), increased LDL (*HR* =2.494, *P* < 0.001), diabetes (*HR* =2.759, *P* < 0.001), hypertension (*HR* =3.471, *P* = 0.037), and CKD stage (*HR* =2.001, *P* = 0.046) were risk factors for CKD progression to ESRD in the overall population. For those without diabetes, only hypoalbuminemia (*HR* =2.938, *P* = 0.030) was a risk factor for CKD progression to ESRD. For those with diabetes, both hypoalbuminemia (*HR* =2.758, *P* = 0.002), the increased level of LDL (*HR* =3.982, *P* < 0.001), and CKD stage (*HR* =3.781, *P* = 0.001) were risk factors for CKD progression to ESRD. The C-index of the final nomograms was 0.760 (*P* < 0.001).

**Conclusions:**

The results from our risk factor model suggest that CKD disease progression can be predicted and early strategic intervention is necessary for CKD patients to avoid renal function deterioration.

## 1. Introduction

Chronic kidney disease (CKD) has been recognized as an important public health issue globally due to CKD patients having an increased risk of end stage renal disease (ESRD). A systematic analysis of the literature showed that the number of CKD patients worldwide reached 697.5 million in 2017, of which China accounted for nearly 19.0% (nearly 132.3 million) [1]. Furthermore, CKD patients often have worse cardiovascular outcomes and higher all-cause mortality [2, 3]. Early screening of high-risk patients could improve the overall costs associated with ESRD prevention [4]. The gradual increase in the cost of dialysis is a concern for CKD patients; however, not everyone with CKD progresses to kidney failure. The prognosis and timing of adverse outcomes in CKD vary from patient to patient [5]. If CKD can be detected early, treatment might slow the decline of kidney function and the potential progression to renal failure.

Diabetes has been a major cause of CKD globally. In the China Kidney Disease Network (CK-NET) 2016 Annual Data Report, almost 13.9% of diabetic patients had CKD [6]. CKD patients, especially complicated with diabetes, might increase cardiovascular events even before end stage kidney disease (ESKD) onset. The main issue related to the increase in cardiovascular events is linked to the vessel microangiopathy damage. Kidney damage in diabetes may be an alert for coronary diseases, which could be even more stressed if diabetic retinopathy is present. Telemedicine application in diabetic retinopathy (DR) has demonstrated efficacy and usefulness in screening diabetic complication [7, 8]. Moreover, the role of multifactorial intervention, as the kidney function drops, becomes always more complicated to perform, as many drugs cannot be used at certain filtrate such as metformin [9, 10]. Therefore, timely screenings for CKD are necessary for early diagnosis and treatment, which improves long-term patient outcomes related to cardiovascular disease (CVD), ESKD, and death [11].

There are, however, limited articles relating risk factors for CKD progression across patients with and without diabetes. Additionally, the significance of findings in a risk factor analysis can differ depending on the specific definition of CKD progression used. The goal of our study was to identify independent risk factors related to CKD progression among those with and without diabetes in hopes of offering personalized approaches for managing patients with advanced CKD and improving access to early prevention and treatment.

## 2. Materials and Methods

### 2.1. Study Population

This study was a retrospective, single-center cohort study. We defined and staged CKD according to the 2012 Clinical Practice Guidelines for CKD Evaluation and Management by Kidney Disease Improving Global Outcomes (KDIGO) [12]. From January 2013 to December 2018, patients with stage 3 and 4 CKD for their first admission to the Nephrology Department of the First Affiliated Hospital of Fujian Medical University were included. We excluded these patients: (1) who were younger than 18 years of age; (2) who had a malignant tumor; (3) who were pregnant; (4) who had a kidney transplant; (5) who had incomplete laboratory or medical data. Included patients were followed up by December 31, 2020. This study was approved by the ethics committee of the First Affiliated Hospital of Fujian Medical University (approval number: [2015]084-2).

### 2.2. Variables and Measurements

For each of the included patients, we collated data on clinical features as follows:

1. Basic demographics, such as sex, age, body mass index (BMI), and primary disease categories
2. Comorbidities, such as diabetes, hypertension, cardiovascular disease, cerebrovascular disease, and gout
3. Complications, such as suffering from AKI, infection, or using nephrotoxic drugs
4. Basic biochemical test values, such as hemoglobin, serum creatinine (SCr), blood urea nitrogen (BUN), albumin (Alb), serum uric acid (UA), cholesterol, low-density lipoprotein (LDL), phosphatase (P), calcium (Ca^+^), sodium (Na^+^), potassium (K^+^), bicarbonate (HCO3^−^), proteinuria, and microalbuminuria/urine creatinine (ACR)
5. Prognosis (whether the patient had started dialysis)

The serum creatinine was measured using an enzymic method though Siemens ADVIA2400. The eGFR was calculated by the CKD-EPI equation [13]. Proteinuria was done using a dry reagent chemistry method for testing urine protein through COMBI UrilyzerAuto, and its test result was as negative or positive. Positive urinary protein had excluded the interference caused by urinary tract infection.

### 2.3. Some Definitions

1. CKD G3a: eGFR 45-59 ml/min·1.73m^2^; CKD G3b: eGFR 30-44 ml/min·1.73m^2^; CKD G4: eGFR 15-29 ml/min·1.73m^2^
2. Anemia: hemoglobin in male <130 g/L, female <120 g/L
3. Hypoalbuminemia: serum albumin <35 g/L
4. Hyperalkaline phosphatase: serum alkaline phosphatase >125 u/L
5. Hypercholesterolemia: total serum cholesterol (TC) >4.14 mmol/L
6. Hyperuricemia: blood uric acid in male >420 mmol/L, female>360 mmol/L
7. Hyperkalemia: serum potassium >5.5 mmol/L
8. Hypocalcemia: serum calcium <2.1 mmol/L
9. Hyperphosphatemia: serum phosphorus >1.45 mmol/L
10. Metabolic acidosis: serum bicarbonate <21 mmol/L
11. Proteinuria: urine routine protein determination is positive
12. Diabetes was identified from the electronic medical record according to the ICD10 code. This diagnosis was verified by use of anti-diabetic medications
13. Hypertension was identified from electronic medical record according to the ICD10 code. This diagnosis was verified use of anti-hypertension medications
14. History of CVD was defined as a previous diagnosis of heart failure, myocardial infarction, valvular heart disease, percutaneous coronary intervention, or bypass grafting
15. History of cerebrovascular disease was defined as a previous diagnosis of cerebral infarction, cerebral hemorrhage, or stroke
16. Nephrotoxic drugs were defined as using non-steroidal anti-inflammatory drugs (NSAIDs), analgesic drugs, aminoglycoside drugs, or contrast medium

### 2.4. Outcomes

The primary outcome was ESRD requiring kidney replacement therapy (KRT). Patients were followed up until December 2020 or the initiation of dialysis. Loss to follow-up, death, or come to the end of a full follow-up process was considered censored data.

### 2.5. Statistical Analysis

For continuous variables, the Shapiro-Wilk test was used to assess whether the data were normally distributed. Data that followed a normal distribution were expressed as means ± standard deviation. Comparisons between groups were carried out by Student's *t*-test. Data that were not normally distributed were expressed as medians and interquartile ranges. Comparisons between groups were carried out using the Kruskal-Wallis test. For categorical variables, comparisons between groups were carried out with the chi-squared test to verify if there were significant differences. Finally, using Kaplan-Meier to plot the survival curve, survival analyses for progression to ESRD were determined using the log-rank test. We determined the univariate factors affecting the development of CKD using the univariate Cox regression analysis. Variables with *P* < 0.05 were entered into the multivariable Cox proportional hazards model. The final model was used to construct nomogram for predicting CKD progression. Statistical significance was defined as *P* < 0.05, and all analysis were performed using R software, version 4.1.2 and SPSS software, version 25.0.

## 3. Results

### 3.1. Study Population Description

From January 2013 to December 2018, we enrolled 309 CKD patients, including 80 cases in stage 3a, 98 cases in stage 3b, and 131 cases in stage 4. Among 309 CKD patients, 141 patients had diabetes and 168 patients did not. The mean age of all CKD patients at the time of study enrollment was 57.86 ± 15.10 years, and 67% were male. The median follow-up time was 25.6 months. There were 81 patients (26.2%) starting dialysis from the total CKD cohort, 52 cases (36.9%) needing dialysis in the CKD with diabetes group, and 29 cases (17.3%) needing dialysis in the CKD without diabetes group.

### 3.2. A Comparison of Clinical Features between Different Groups

Table 1 shows the differences in demographic variables between the CKD subgroups. We found that there were statistically significant differences in these demographic variables, such as age, BMI, the follow-up time, eGFR at first, CR at first, BUN, HGB, Alb, serum phosphorus, ACR, HbA1, eGFR at end point, and CR at end point, indicating that increased age and elevated body mass index were more common in CKD patients with diabetes when compared to those without diabetes. What's more, in the group of CKD patients with diabetes, the elevation in levels of CR, BUN, serum phosphorus, ACR, and HbA1 was more pronounced than those without diabetes. The levels of HGB, eGFR, Alb, and the follow-up time were significantly lower in CKD patients with diabetes when compared to those without diabetes. The difference in clinical characteristics seen between the CKD subgroups is shown in Table 2. Among these differences, only hypertension showed a statistically significant difference between the CKD subgroups, illustrating that CKD patients with diabetes experienced hypertension more often than those without diabetes. There were no statistically significant differences in proteinuria, history of AKI, infection, cardiovascular disease, cerebrovascular disease, or gout between the CKD subgroups.

### 3.3. Survival Curve by the Kaplan-Meier Analysis

**Figure 1:**
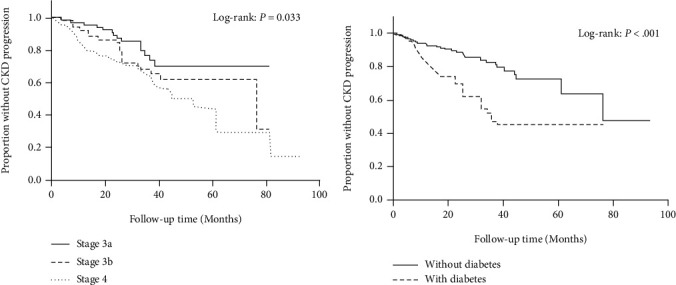
The Kaplan-Meier survival plot of CKD progression by CKD stages (a) and diabetic status (b).

We furtherly performed the survival curve by the Kaplan-Meier analysis. The survival curves of patients at different CKD stages G3a, G3b, and G4 are shown in Figure 1(a) (*P* = 0.033). The Kaplan-Meier analysis revealed that CKD G3b and G4 patients had a higher possibility of progression to ESRD when compared with CKD G3a.

Figure 1(b) shows the survival curves of patients between CKD subgroups. The CKD with diabetes group showed significantly higher risk of progress to ESRD when compared to those without diabetes (*P* < 0.001).

### 3.4. Prediction Model Performance for All CKD Patients

#### 3.4.1. Univariate Analysis

Table 3 shows the univariate variable analysis of all the clinical features. We found that serum creatinine, eGFR, hypoalbuminemia, BUN, the increased level of LDL, hypocalcemia, diabetes, hypertension, and CKD stage were risk factors for CKD progression to ESRD.

#### 3.4.2. Multivariate Analysis

**Figure 2:**
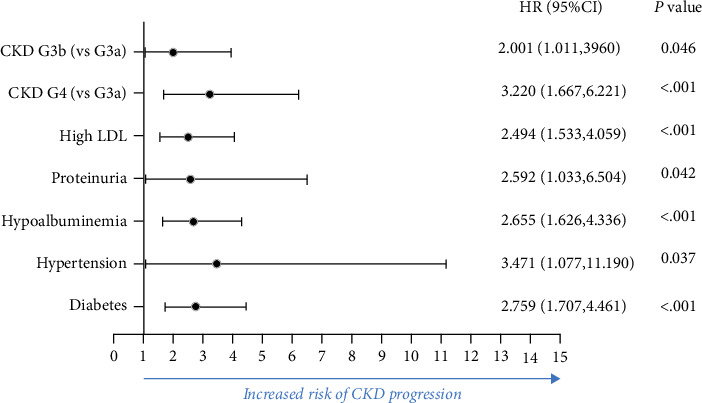
Baseline characteristics associated with CKD progression using multivariate analysis.

Table 4 presents the multivariable analysis of all CKD patients. We found that hypoalbuminemia, proteinuria, the increased level of LDL, diabetes, hypertension, and CKD stage were risk factors for CKD progression to ESRD (Figure 2). Our study revealed that CKD patients with diabetes had a 2.759 times increase (95% CI: 1.707-4.461) in progression to dialysis when compared to those without diabetes.

When multivariate Cox analyses were performed among CKD subgroups, the results were different which are presented in Table 5. Specifically, for those without diabetes, only hypoalbuminemia was observed as a risk factor for CKD progression to ESRD. For those with diabetes, hypoalbuminemia, increased LDL, and CKD stage were determined to be risk factors for CKD progression to ESRD.

### 3.5. Construction and Calibration of Nomogram

**Figure 3:**
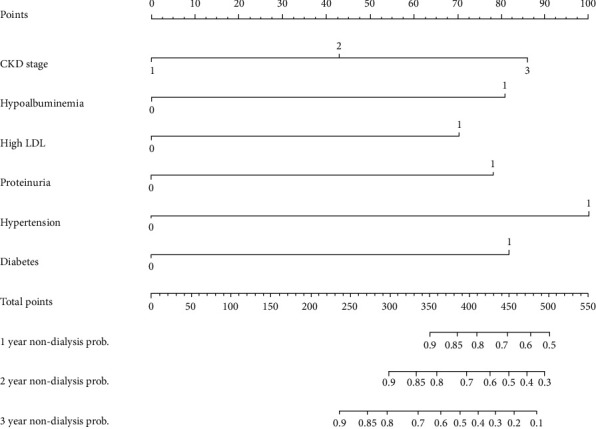
Prognostic nomograms. To use the nomogram, based on significant prognostic variables, draw upward lines from each variable axis to the points and calculate the sum of these points. And draw a downward line from total point axis to the non-dialysis axis to get the likelihood of 1 years non-dialysis prob., 2 years non-dialysis prob. and 3 years non-dialysis prob.

**Figure 4:**
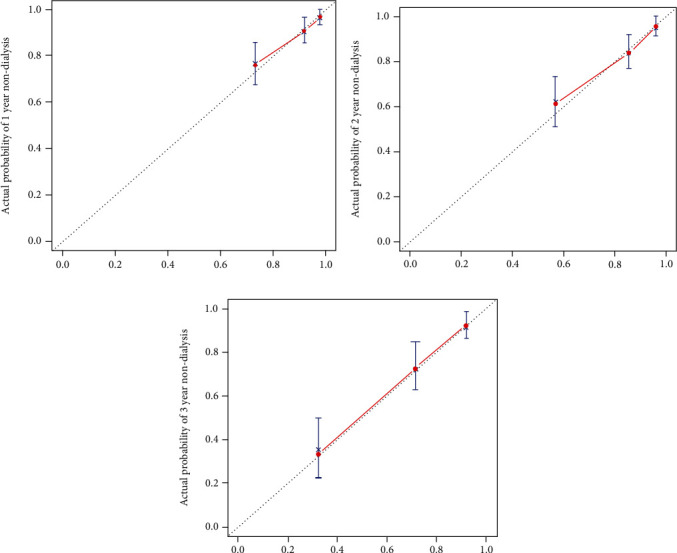
Nomogram-predicted probability of 1 year (a)/2 years (b)/3 years (c) non-dialysis patient status.

Our nomogram included significant prognostic variables in the model for non-dialysis probability of CKD patients at 1 year, 2 years, and 3 years and is presented in Figure 3. The C-index for the final nomograms was 0.760 (*P* < 0.001). The highest point was assigned to hypertension and diabetes in the nomogram, while proteinuria and LDL level played minor roles. The calibration curve revealed high consistency between predicted and actual 1-year, 2-year, and 3-year non-dialysis probability of CKD patients (Figures 4(a)–4(c)).

## 4. Discussion

Significant progress has been made in the prediction of CKD progression using available clinical data in recent years [14]. Our findings showed that hypoalbuminemia, diabetes, CKD stage, increased LDL, proteinuria, and hypertension were risk factors for CKD progression for all CKD patients. For the subgroup of CKD patients with diabetes, the risk factors were CKD stage, increased LDL, and hypoalbuminemia, but only hypoalbuminemia was considered a risk factor for CKD progression in CKD patients without diabetes. Therefore, we found that hypoalbuminemia was an independent risk factor for CKD progression regardless of whether the patients had diabetes mellitus, indicating the importance of serum albumin level for CKD progression. This finding also indicates that patients should be monitored for malnutrition as part of efforts to delay CKD progression. Since the proportion of patients with proteinuria and hypertension in the diabetic group was very high (87.9% and 93.6%, respectively), after stratification of diabetes, hypertension and proteinuria were no longer risk factors for CKD progression. We assume that hypertension and proteinuria are associated with diabetes. This finding indirectly reflects the great impact of diabetes on CKD progression, and it is consistent with our clinical observation that diabetic kidney disease often progresses to ESRD more quickly than other CKD disease. Increased LDL was found to be a risk factor for CKD progression only in CKD patients with diabetes, indicating that diabetes patients are more prone to having glucose and lipid metabolism disorders [15], and it is necessary to strengthen the monitoring and intervention of dyslipidemia to help delay the progression of CKD in these patients.

Hypoalbuminemia remained an independent risk factor for CKD progression in our study no matter with or without diabetes. Serum albumin has been shown to represent nutritional status and chronic inflammation in CKD patients [16]. A study including 4061 CKD 3b-5 patients found that albumin levels were a significant risk factor for initiating dialysis [17]. Another study indicated that a lower level of serum albumin predicted a higher risk of kidney failure, which was consistent with our study [18]. A study demonstrated that metabolic acidosis can lead to hypoalbuminemia, which contributes to CKD progression [19]. For low molecular weight proteins (<20 kDa), they are not recycled in the kidney but returned to the blood supply after being fully degraded. Hypoalbuminemia occurs when chronic injured kidney cannot retrieve nephrotic protein excretion [20]. Hypoalbuminemia is the result of an inflammatory process [21]. Serum albumin might contribute to the development of CKD through its antioxidant and anti-inflammatory effects [22]. Current strategies for correcting hypoalbuminemia in CKD patients include controlling systemic inflammation, improving immune function, optimizing diet, and managing other comorbidities [23].

In terms of diabetic comorbidity, our study found that CKD patients with diabetes had a 2.759 times increased risk (95% CI: 1.707-4.461) of progressing to dialysis, compared to those without diabetes. These findings are similar to that of a large-scale population-based cohort study, which found that diabetic subjects reached kidney failure about twice as rapidly as non-diabetic subjects [24]. A global meta-analysis (including 28 cohorts; 185024 patients) also found that diabetes was a risk factor for all outcomes, such as KRT, CVD, and death [25]. Other previous studies have also proven that diabetes increases the risk of CKD progression to dialysis [26–28]. Recent advances in molecular and genetic technology have presented new opportunities for biomarker discovery and the development of personalized treatment options [29]. Diagnosis and disease progression prediction for CKD have improved in recent years. It is recommended that diabetic patients undergo extensive screening to help diagnose and treat CKD at an early stage [30]. Concerning treatment, studies have found that multifactorial intensive therapy aimed at main cardiovascular risk factors such as hypertension, glycol-metabolic control, and dyslipidemia can reduce the risk of cardiovascular events and mortality in high-risk DKD patients [10, 31]. The current study also had demonstrated that SGLT2 inhibitors and GLP-1 receptor agonists not only had strong glucose-lowering effects but also can prevent renal damage and the onset of chronic kidney disease, playing a critical role in DKD progression [32]. One of the limitations of our study was that we were not able to include these data. In future study, it is necessary to include SGLT2 inhibitors as a treatment to evaluate its efficiency to slowing CKD progression.

Nephrologists have previously characterized patients with an advanced CKD stage as being a high-risk population for needing KRT, and our study also found that CKD G3b and G4 had a higher risk for progress to ESRD when compared to CKD G3. The Chronic Renal Insufficiency Cohort (CRIC) study also came to a similar conclusion, finding that more advanced baseline CKD stage was associated with a higher risk of clinical events and faster eGFR decline [33]. Another study found that patients with CKD G4+ largely represented a high-risk CKD population requiring advanced care and decision-making by specialized nephrologists [34].

Besides CKD stage, other risk factors also were associated with needing KRT. Our results show an independent association between elevated levels of LDL and needing KRT in CKD patients. A retrospective study that included 14,510 male workers found increased levels of LDL were associated with the development of CKD and eGFR decline in young to middle-aged working men without hypertension and/or diabetes [35]. By contrast, another study showed that lowering LDL by 1 mmol/L did not slow kidney disease progression within 5 years in a wide range of patients with CKD [36]. This may indicate that LDL is a controversial risk factor for CKD progression. Chronic inflammation and oxidative stress cause endothelial dysfunction, which is an important risk factor for atherosclerosis [37]. Increased LDL can trigger pro-inflammatory, oxidative stress, and pro-atherogenic processes, which induce mitochondrial dysfunction and cellular damage, and contribute to the progression of kidney damage [38]. Diabetic dyslipidemia is caused by metabolic dysregulation of TG-rich lipoproteins (TRL) in an insulin-dependent manner, and this process is thought to be exacerbated by CKD progression [15]. Future study is needed to prove the effects of lowering LDL on the progression of CKD.

This study found that increased proteinuria also increases the risk of dialysis by 2.592 times (95% CI: 1.033-6.504). It has been proven that proteinuria is an important factor in CKD progression [26, 39, 40]. Regardless of diabetic status, the strongest independent predictors for fast CKD progression include proteinuria [41]. As we know, blockade of the renin-angiotensin II (Ang II) system by AT1 blockers (ARBs) and angiotensin-converting enzyme inhibitors (ACEIs) retards the progression of CKD by reducing proteinuria [42]. Albuminuria and tubular atrophy are also risk factors for the progression of CKD to ESRD, and kidney proximal tubule lipoapoptosis is caused by dysregulation of fatty acid transporter-2 (FATP2), which may be an appropriate molecular target for the treatment of CKD [43]. Proteinuria represents a significant prognostic factor for onset and progression of DKD and CVD, reducing proteinuria can improve the cardio-renal outcomes in diabetic patients [44]. On the other hand, CKD is one of the strongest risk factors for the development of CVD. For CKD patients, the risk of developing CVD surpasses the risk of reaching ESRD, so cardiovascular death rather than ESRD is the leading cause of death [45]. Novel therapies to decrease the risk of CVD in CKD are in clinical development, raising the hope that cardiovascular risk in CKD patients may be modifiable in the future [46].

In our analysis, hypertension was associated with an increased risk of requiring KRT. Similarly, a prospective cohort study in Korea found that 90.6% of their CKD cohort also had hypertension [47]. One study indicated that diabetic CKD patients have a J-shaped relationship between systolic blood pressure and renal outcomes when compared with non-diabetic CKD patients [48]. Another study also demonstrated that the risk of diabetic renal function impairment in the first decade after being diagnosed with diabetes mellitus was correlated with high variability of visit-to-visit systolic and diastolic blood pressure [49]. Our results were consistent with a recently published study, which found hypertension was an important risk factor for CKD progression to dialysis [50]. A cross-sectional study including 1814 CKD patients found that CKD patients with hypertension aged 65 or older, or with severe albuminuria or proteinuria, all of which put patients at risk of kidney disease, were found to have higher rates of uncontrolled BP [51]. It is important to keep blood pressure within the normal range. Optimal blood pressure control provides significant but incomplete renal protection [52].

This study has several strengths. Firstly, we investigated CKD G3-G4 patients with or without diabetes and compared the differences between them in the progression of CKD to ESRD so as to help identify the role of diabetes in the progression of CKD. Secondly, we established a predictive model to generate accurate risk factors. Finally, we selected factors that can be acquired easily during routine clinical assessments. Consequently, our final model has strong potential applicability for doctors to identify CKD patients who are at high risk of accelerated renal function loss when making important clinical decisions.

Nevertheless, it should be noted that some limitations exist in our study. First of all, it was a retrospective single-center study covering only a small population that might result in a population selection bias. What's more, most CKD G3 patients were followed up at the outpatient department, so CKD G3 patients included in this study were likely to have other complications, which may cause selection bias, too. In addition, there was a statistical difference in the follow-up time between CKD patients with and without diabetes, which might have interfered with the results. This may be due to the fact that the end point events often occurred earlier in the CKD patients with diabetic group. Finally, there was some missing data during collection; hence, we used average values to replace missing data. Therefore, further multi-center studies will be needed to confirm the validity of our findings in future studies.

## 5. Conclusion

We developed a prediction model that can be used to predict the probability of progression to dialysis in CKD patients with and without diabetes. Our results suggest that early strategic intervention is necessary for CKD stage G3 and G4 patients to avoid renal function deterioration. We suggest interventions to help prevent progression of CKD, such as strict control of blood glucose, blood pressure, albumin, LDL, and proteinuria, regardless of diabetic status. These findings should encourage nephrologists to assess traditional risk factors in CKD G3 to G4 patients and offer interventions to reduce exposure to avoidable risks.

## Figures and Tables

**Table 1:** Basic clinical characteristics of all patients and diabetic patients.

Variable	All patients (*N* =309)	Diabetes status		*t*/*H* value	*P* value^a^
		Without diabetes (*N* =168)	With diabetes (*N* =141)		
Age (years)	57.86 ± 15.10	54.12 ± 17.11	62.31 ± 10.75	16.686	<0.001
BMI (kg/m^2^)	23.88 (21.33, 25.52)	23.36 (20.70, 24.90)	24.70 (22.34, 25.93)	10.579	0.001
Follow-up time (m)	25.67 (12.45, 37.02)	28.55 (18.81, 39.38)	23.90 (9.42, 33.62)	9.183	0.002
eGFR at first (mL/min/1.73m^2^)	33.56 (24.11, 45.63)	34.14 (25.56, 45.47)	32.55 (20.63, 45.83)	4.596	0.032
CR at first (*μ*mol/L)	185.05 ± 61.78	177.38 ± 50.46	194.19 ± 72.15	-2.401	0.017
Urea nitrogen (mmol/L)	11.00 (7.70, 13.86)	10.23 (7.45, 13.23)	11.40 (8.06.14.45)	5.389	0.020
Hemoglobin (g/L)	107.74 ± 22.60	113.66 ± 22.28	100.68 ± 20.96	5.239	<0.001
Serum albumin (g/L)	35.20 (29.75, 39.20)	36.80 (31.90, 39.80)	34.00 (28.75, 37.60)	7.593	0.006
Uric acid (*μ*mol/L)	431.82 ± 102.42	430.56 ± 100.26	433.32 ± 105.27	-0.236	0.814
ALP (U/L)	68.00 (55.00, 87.00)	67.00 (55.00, 84.00)	71.00 (56.50, 91.00)	2.323	0.128
Triglyceride (mmol/L)	2.04 (1.33, 4.21)	1.84 (1.29, 4.20)	2.14 (1.37, 4.24)	1.129	0.288
LDL (mmol/L)	2.83 (1.95, 3.71)	2.91 (2.16, 3.70)	2.71 (1.83, 3.72)	1.771	0.183
Calcium (mmol/L)	2.16 (2.04, 2.26)	2.16 (2.06, 2.26)	2.16 (2.01, 2.26)	0.072	0.789
Phosphorus (mmol/L)	1.22 (1.09, 1.39)	1.20 (1.06, 1.34)	1.26 (1.12, 1.45)	4.483	0.034
Potassium (mmol/L)	4.29 ± 0.64	4.28 ± 0.62	4.31 ± 0.67	-0.419	0.676
Bicarbonate (mmol/L)	23.68 ± 3.51	23.47 ± 3.37	23.92 ± 3.67	-1.120	0.263
ACR (mg/g)	1064.39 (448.82, 3522.24)	1166.46 (282.99, 2477.71)	2327.14 (766.48, 4152.06)	11.667	0.001
HbA1c (%)	6.20 (5.50,7.20)	5.75 (5.47,6.40)	7.20 (6.30,8.50)	-6.636	<0.001
eGFR at end point (mL/min/1.73m^2^)	19.05 (8.20, 40.56)	23.45 (11.74,47.20)	12.42 (6.60,25.08)	4.658	0.001
CR at end point (*μ*mol/L)	391.2 ± 290.8	351.4 ± 288.4	473.5 ± 279.6	-3.512	0.001

Abbreviations: BMI: body mass index; CR: creatinine; eGFR: estimated glomerular filtration rate; ALP: alkaline phosphatase; LDL: low-density lipoprotein; ACR: microalbuminuria/urine creatinine; ^a^*P*-value for comparison across those with and without diabetes. The *t* test was used to compare the normally distributed variables (serum creatinine, hemoglobin, uric acid, serum potassium, and bicarbonate). The Kruskal-Wallis test was used to compare the non-normally distributed variables (age, BMI, follow-up time, eGFR, urea nitrogen, serum albumin, uric albumin, ALP, triglyceride, LDL, serum calcium, and serum phosphorus).

**Table 2:** Baseline clinical characteristics of all patients and diabetic patients.

Variable	All patients (*N* =309)	Diabetes status		*χ* ^2^ value	*P* value^a^
		Without diabetes (*N* =168)	With diabetes (*N* =141)		
Gender (male), *N* (%)	207 (67.0%)	113 (67.3%)	94 (66.7%)	0.012	0.912
CKD stages, *N* (%)				0.636	0.727
G3a	80 (25.9%)	44 (26.2%)	36 (25.5%)		
G3b	98 (31.7%)	56 (33.3%)	42 (29.8%)		
G4	131 (42.4%)	68 (40.5%)	63 (44.7%)		
Proteinuria, *N* (%)	268 (87.3%)	146 (86.9%)	122 (87.8%)	0.051	0.821
History of AKI, *N* (%)	23 (7.5%)	10 (6.0%)	13 (9.3%)	1.228	0.268
History of infection, *N* (%)	85 (27.5%)	43 (25.6%)	42 (29.8%)	0.676	0.411
Nephrotoxic drugs, *N* (%)	31 (10.1%)	21 (12.7%)	10 (7.1%)	2.531	0.112
ACEI/ARB, *N* (%)	114 (36.9%)	59 (35.1%)	55 (39.0%)	0.498	0.480
Hypertension, *N* (%)	269 (87.1%)	137 (81.5%)	132 (93.6%)	9.909	0.002
Cardiovascular disease, *N* (%)	92 (29.8%)	49 (29.2%)	43 (30.5%)	0.065	0.799
Cerebrovascular disease, *N* (%)	23 (7.4%)	11 (6.5%)	12 (8.5%)	0.429	0.513
Gout, *N* (%)	39 (12.7%)	24 (14.4%)	15 (10.6%)	0.963	0.326
Start dialysis, *N* (%)	81 (26.2%)	29 (17.3%)	52 (36.9%)	15.253	<0.001

Abbreviations: CKD: chronic kidney disease; AKI: acute kidney injury. ^a^*P*-value for comparison across those with and without diabetes. The chi-squared test was used to compare the categorical variables.

**Table 3:** Univariate analysis of factors affecting the development of chronic kidney disease.

Variable	*B* value	HR (95% CI)	*P* value
Basic demographic variables			
Gender	0.248	1.281 (0.784, 2.094)	0.322
Age	-0.010	0.990 (0.976, 1.004)	0.154
BMI	-0.003	0.997 (0.933, 1.064)	0.921
Laboratory tests			
Serum creatinine	0.006	1.006 (1.003, 1.010)	<0.001
eGFR	-0.026	0.974 (0.957, 0.992)	0.005
Anemia	0.357	1.429 (0.795, 2.569)	0.233
Hypoalbuminemia	1.240	3.457 (2.130, 5.610)	<0.001
Proteinuria	1.089	2.973 (1.197, 7.381)	0.019
ACR			
A2 (vs A1)	0.100	1.105 (0.100, 12.266)	0.935
A3 (vs A1)	2.238	9.377 (1.296, 67.831)	0.027
ALP	0.003	1.003 (0.999, 1.006)	0.114
Urea nitrogen	0.100	1.105 (1.062, 1.151)	<0.001
Hyperuricemia	-0.340	0.712 (0.454, 1.116)	0.139
Hypertriglyceridemia	0.297	1.346 (0.866, 2.091)	0.186
High LDL	0.682	1.979 (1.261, 3.106)	0.003
Hypocalcemia	0.517	1.677 (1.070, 2.628)	0.024
Hyperphosphatemia	0.190	1.209 (0.716, 2.042)	0.478
Hyperkalemia	-0.049	0.952 (0.300, 3.020)	0.934
Metabolic acidosis	-0.001	0.999 (0.570, 1.753)	0.998
Clinical characteristics			
CKD stages			
G3b (vs G3a)	0.459	1.583 (0.826, 3.035)	0.167
G4 (vs G3a)	0.780	2.182 (1.186, 4.011)	0.012
History of AKI	0.015	1.015 (0.409, 2.516)	0.974
History of infection	0.075	1.078 (0.660, 1.759)	0.765
Nephrotoxic drugs	-0.535	0.586 (0.237, 0.450)	0.247
ACEI/ARB	0.089	1.093 (0.698, 1.710)	0.698
Hypertension	1.506	4.508 (1.421, 14.296)	0.011
Diabetes	1.016	2.763 (1.750, 4.362)	<0.001
Cardiovascular disease	0.091	1.095 (0.686, 1.750)	0.703
Cerebrovascular disease	0.228	1.256 (0.578, 2.731)	0.564
Gout	-0.277	0.758 (0.378, 1.519)	0.434

Abbreviations: BMI: body mass index; eGFR: estimated glomerular filtration rate; ACR: microalbuminuria/urine creatinine, A1: UACR<30 mg/g; A2: UACR 30~300 mg/g; A3: UACR>300 mg/g; ALP: alkaline phosphatase; LDL: low-density lipoprotein; CKD: chronic kidney disease; AKI: acute kidney injury.

**Table 4:** Multivariate analysis of factors affecting the progression of CKD.

Variable	*B* value	HR (95% CI)	*P* value
Hypoalbuminemia	0.977	2.655 (1.626, 4.336)	<0.001
Proteinuria	0.953	2.592 (1.033, 6.504)	0.042
High LDL	0.914	2.494 (1.533, 4.059)	<0.001
Diabetes	1.015	2.759 (1.707, 4.461)	<0.001
Hypertension	1.245	3.471 (1.077, 11.190)	0.037
CKD stages			
G3b (vs G3a)	0.694	2.001 (1.011, 3.960)	0.046
G4 (vs G3a)	1.169	3.220 (1.667, 6.221)	<0.001

Abbreviations: LDL: low-density lipoprotein; CKD: chronic kidney disease.

**Table 5:** Multivariate analysis of factors affecting the CKD progression among patients with or without diabetes.

Variable	Without diabetes			With diabetes		
	*B* value	HR (95% CI)	*P* value	*B* value	HR (95% CI)	*P* value
Hypoalbuminemia	1.078	2.938 (1.113, 7.758)	0.030	1.014	2.758 (1.473, 5.165)	0.002
High LDL	0.119	1.127 (0.460, 2.757)	0.794	1.382	3.982 (2.094, 7.574)	<0.001
CKD stages						
G3b (vs G3a)	0.569	1.767 (0.469, 6.659)	0.400	0.887	2.428 (1.070, 5.511)	0.034
G4 (vs G3a)	1.232	3.429 (0.979, 12.011)	0.054	1.313	3.718 (1.681, 8.225)	0.001

Abbreviations: LDL: low-density lipoprotein; CKD: chronic kidney disease.

## Data Availability

The data that support the findings of this study are available from the corresponding author upon reasonable request.

## Abbreviations

CKD: Chronic kidney disease
ESRD: End stage renal disease
KDIGO: Kidney Disease: Improving Global Outcomes
BMI: Body mass index
CVD: Cardiovascular disease
SCr: Serum creatinine
BUN: Blood urea nitrogen
ALB: Albumin
UA: Serum uric acid
LDL: Low-density lipoprotein
P: Phosphatase
Ca^+^: Calcium
Na^+^: Sodium
K^+^: Potassium
HCO3^−^: Bicarbonate
ACR: Microalbuminuria/urine creatinine
NSAID: Non-steroidal anti-inflammatory drug
KRT: Kidney replacement therapy
DKD: Diabetes kidney disease.
